# Optical coherence tomography assessment of disease activity in cryopyrin‐associated periodic syndrome

**DOI:** 10.1111/ene.16301

**Published:** 2024-04-16

**Authors:** E. Mulazzani, L. Böhm, T. Christmann, M. Krumbholz, T. Kümpfel, J. Havla

**Affiliations:** ^1^ Institute of Clinical Neuroimmunology LMU University Hospital, LMU Munich Munich Germany; ^2^ Department of Neurology and Pain Treatment, Immanuel Klinik Rüdersdorf University Hospital of the Brandenburg Medical School Theodor Fontane Rüdersdorf bei Berlin Germany; ^3^ Faculty of Health Sciences Brandenburg Brandenburg Medical School Theodor Fontane Rüdersdorf bei Berlin Germany; ^4^ Department of Neurology and Stroke University Hospital of Tübingen Tübingen Germany

**Keywords:** autoinflammation, NLRP3 variants, OCT

## Abstract

**Background and purpose:**

Cryopyrin‐associated periodic syndrome is a rare autoinflammatory disease caused by gain‐of‐function mutations or variants in the *NLRP3* gene. Clinically, patients suffer from a broad spectrum of both systemic and neurological symptoms. The aim of this study was to determine whether systemic inflammation demonstrated by serum amyloid A (SAA) elevation is associated with neuroinflammation assessed by optical coherence tomography (OCT).

**Methods:**

Thirty eyes of 15 patients with NLRP3 low penetrance mutations (PwNLRP3) and 20 eyes of 10 age‐ and sex‐matched healthy controls were examined by spectral‐domain OCT as part of routine clinical care. All retinal layers and clinical features were evaluated.

**Results:**

At baseline no significant retinal neuroaxonal inflammation or degeneration was observed in all measured retinal layers amongst PwNLRP3 compared with healthy controls. In a pooled analysis of all individual OCT time points a significant difference regarding the macular retinal nerve fibre layer was detected. Increased levels of SAA showed a positive association with averaged combined outer plexiform layer and outer nuclear layer volumes (*ρ* < 0.0001, *r* ^2^ = 0.35).

**Conclusion:**

In cryopyrin‐associated periodic syndrome increased combined outer plexiform layer and outer nuclear layer volumes are mirrored by SAA increase, an acute phase reactant indicating systemic inflammation. Our findings identify OCT as a candidate biomarker to monitor subclinical neuroinflammation and to assess disease activity in PwNLRP3.

## INTRODUCTION

Cryopyrin‐associated periodic syndromes (CAPSs) comprise a group of rare, yet treatable hereditary autoinflammatory diseases caused by gain‐of‐function mutations/variants in the NLRP3 inflammasome [1]. Oligomerization of mutated NLRP3 leads to excessive interleukin‐1β (IL‐1β) levels, thus resulting in multisystemic inflammation across all organs and elevated acute phase reactants [2]. Neurological deficits in CAPS range from aseptic meningitis/meningoencephalitis, cerebral atrophy, hydrocephalus, sensorineural deafness to vision loss and are often irreversible and detrimental [3]. It was recently shown that two‐thirds of patients with *NLRP3* low penetrance variants (PwNLRP3) are at high risk to develop severe neuroinflammation; however, neurologists are still lacking biomarkers to assess disease activity [4, 5]. Along that line, this study aimed to investigate the relationship between systemic inflammation and inflammation of the central nervous system (CNS) in PwNLRP3 by retinal optical coherence tomography (OCT) usage.

## METHODS

### Patients

**FIGURE 1:**
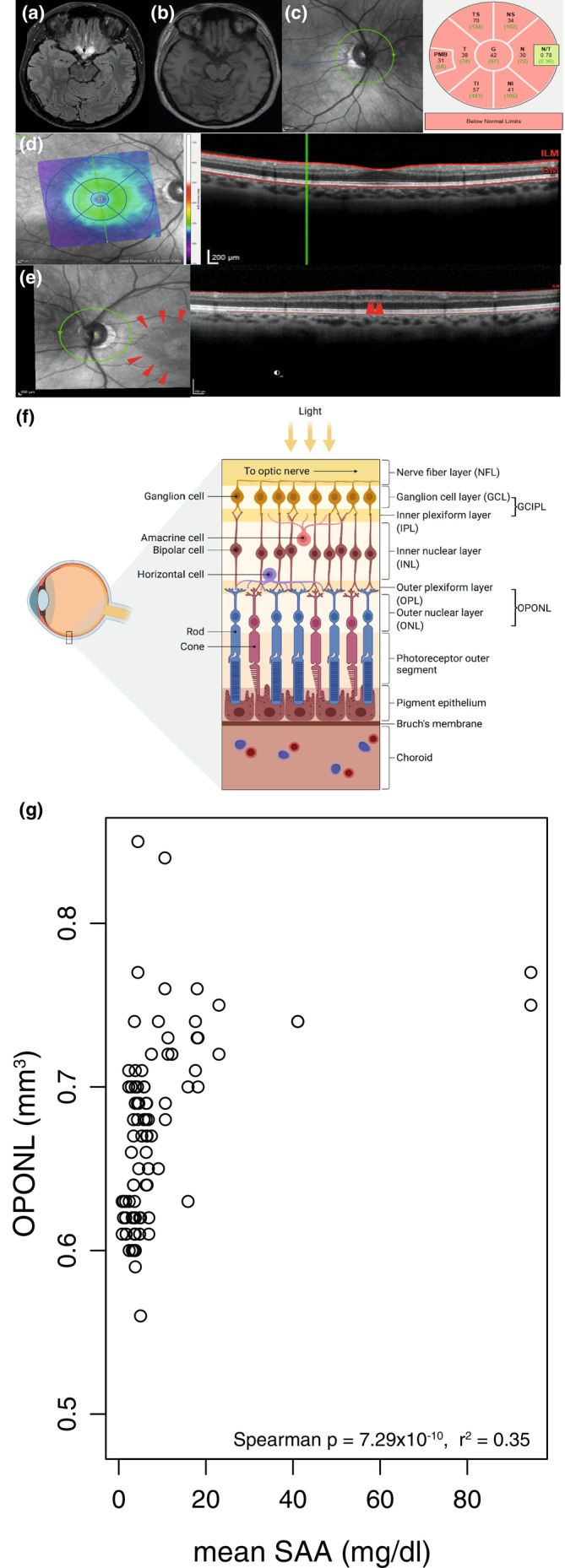
Cerebral magnetic resonance imaging and OCT images of a PwNLRP3 was diagnosed with relapsing inflammatory optic neuropathy due to recurrent episodes of ON in both eyes at the age of 43. Not long after her first neurological consultation she started manifesting pain and oedema in her left Achilles tendon. Clinical progression over the following years, typically accompanied by signs of inflammation in both blood (elevated SAA and CRP levels) and CSF (pleocytosis and CSF‐specific oligoclonal bands) led to further diagnostics. The systemic workup was negative for immunological markers (aquaporin‐4 antibodies, MOG‐IgG, ANA, c‐ANCA and p‐ANCA, s‐IL‐2 receptor) or infectious aetiologies (HSV, VZV, CMV, measles, rubella and lues IgG, borrelia and toxoplasma IgG/IgM). Genetic analyses revealed a homozygous low penetrance variant in the *NLRP3* gene compatible with CAPS. (a) Axial fluid attenuated inversion recovery sequence showing massive swelling of the chiasma‐pituitary region and both nervi optici. (b) Gadolinium enhanced T1‐weighted images demonstrating contrast enhancement involving both nervi optici. (c) OCT images showing the ring scan and (d) the macula scan of the index patient. Global subtotal atrophy of the pRNFL is shown in (c) as well as morphological evidence of bilateral volume reduction (TMV) (d). (e) OCT images of the inner nuclear layer indicating MME (red arrows). The prevalence of MME in a CAPS cohort is unknown; in our cohort of PwNLRP3 MME could be detected in 6.7% of the eyes and only in the INL. Overall, the findings are consistent with a pronounced neuroaxonal retinal degeneration. In this patient, there was only one follow‐up OCT examination at an interval of 6 months. In this short course, no further retinal neuroaxonal degeneration was observed. (f) Retinal layers adapted from ‘Structure of the retina’ by BioRender.com (2020). Retrieved from https://app.biorender.com/biorender‐templates. (g) Correlation of OPONL and mean SAA levels (assessed at the OCT examination). SAA levels of >5.0 mg/L were considered abnormal. Spearman's rank correlation coefficient *r* and exact *p* values are reported.

For this analysis clinical, laboratory (C‐reactive protein [CRP], serum amyloid A [SAA], leucocyte counts) and OCT data collected between 2014 and 2019 were retrospectively included. Clinical details of an index patient are shown in Figure 1a‐e. SAA values >5.0 mg/L were considered abnormal and were routinely determined at the Institute of Laboratory Medicine, LMU University Hospital, Munich, during the identical time point of OCT assessment. Inclusion criteria encompassed established diagnosis of PwNLRP3, the genetic verification of mutation or variants in the *NLRP3* gene and the presence of follow‐up OCT imaging data. Healthy volunteers from the Institute of Clinical Neuroimmunology were recruited to serve as controls for this study. The study was approved by the local ethics committee of the LMU Munich (project no. 600‐15 and 427‐14). Written informed consent was obtained from each participant according to the Declaration of Helsinki.

### Optical coherence tomography

Optical coherence tomography examination was performed using a SD‐OCT (Spectralis, Heidelberg Engineering, Heidelberg, Germany) with automatic real time (ART) function for image averaging. One experienced rater checked all scans for sufficient quality and segmentation errors and corrected, if required. The volumes of the peripapillary retinal nerve fibre layer (pRNFL), macular retinal nerve fibre layer (mRNFL), combined ganglion cell and inner plexiform layer GCIPL, the inner nuclear layer (INL), outer plexiform layer and outer nuclear layer (OPONL) and the total macular volume (TMV) were analysed (Figure 1f). Calculation of macular layers is given for a 3 mm diameter cylinder around the fovea from a macular volume scan (20° × 20°, 25 vertical B‐scans, ART ≤49). The pRNFL was measured with an activated eye tracker using 3.4 mm ring scans around the optic nerve (12°, 1536 A‐scans, ART ≤100). Segmentation of all layers was performed semi‐automatically using software provided by the OCT manufacturer (Eye Explorer 1.9.10.0 with viewing module 6.3.4.0, Heidelberg Engineering). In addition, the presence of microcystic macular oedema (MME) was examined in the INL and OPONL. MME was defined as the presence of cystic microlesions on at least two adjacent B‐scans. OCT data in this study are reported according to the APOSTEL and OSCAR‐IB recommendations [6, 7, 8].

### Statistical analysis

Group differences were assessed with the non‐parametric Mann–Whitney *U* test. Frequencies and percentages were used as descriptive statistics for categorical variables. Correlations were calculated using the Spearman's rank correlation coefficient *r*. Additionally, cross‐sectional differences of OCT values between groups were analysed pairwise by generalized estimating equation (GEE) models to account for inter‐eye within‐patient correlations of monocular measurements. Analyses of all OCT time points were performed by a linear mixed effects model. Mixed effects models and GEE analysis were both adjusted for relevant confounders including age and sex. Data of the GEE and linear mixed effect models are not shown but are made available upon request. All tests and graphical representations were performed with R V.3.3.1 (http://www.R‐project.org). Statistical significance was established at *p* < 0.05, and all results were interpreted in the context of an exploratory analysis without adjusting for multiple comparisons.

## RESULTS

### Patient cohort, control cohort and genetics

Fifteen PwNLRP3 (four males, 11 females; 50 ± 13 years at study baseline) and 10 healthy controls (HCs) (three males, seven females; 38 ± 13 years) were included in the study. All patients were Caucasian adults. NLRP3 variants comprised the low penetrance substitution V198M (*n* = 2) and Q703K (*n* = 13). Ten patients (*n* = 10; 67%) fulfilled the diagnostic criteria for CAPS [9].

### Clinical features

The median age at symptom onset was 33 ± 14 years. Most patients showed a recurrent (*n* = 9; 60%) disease course with flares. Organ involvement included the musculoskeletal system (*n* = 10; 67%), eyes (uveitis, *n* = 5; 33%), skin (*n* = 3; 20%), gastrointestinal tract (*n* = 3; 20%) and central nervous system (CNS) (*n* = 15; 100%). Regarding CNS manifestation beyond optic neuritis (ON) (*n* = 10; 67%), three patients suffered from recurrent aseptic meningoencephalitis and one from cerebral vasculitis with ischaemic strokes. Overall, 12/15 patients (80%) showed intermittent elevated levels of acute phase reactants (SAA [*n* = 10; 67%], CRP [*n* = 2; 13%] and leucocytosis [*n* = 1; 7%]) without signs of infection. Lumbar puncture was performed in all patients. Cerebrospinal fluid (CSF) abnormalities (inflammatory CSF syndrome with pleocytosis and/or detection of oligoclonal bands) were found in 11/15 patients (73%). Magnetic resonance imaging data were available in all patients. 10/15 patients showed parenchymal lesions (67%). Treatment with anti‐IL‐1 inhibitors in nine patients (*n* = 9; 60%) was applied.

### Group differences at baseline

Taking an explorative study approach, group differences at baseline between PwNLRP3 compared to HCs without consideration of the current disease activity were first analysed. At baseline, there was no significant difference between various macular and peripapillary layers (global pRNFL, *p* = 0.36; pRNFL papillo‐macular bundle, *p* = 0.86; pRNFL nasal, *p* = 0.43; pRNFL temporal, *p* = 0.62; TMV, *p* = 0.93; mRNFL, *p* = 0.29; INL, *p* = 0.12; GCIPL, *p* = 0.37; OPONL *p* = 0.84) (see Table 1).

**TABLE 1:** OCT results of HC and NLRP3 variants at baseline and follow‐up.

	Baseline					Follow‐up				
	Healthy controls		NLRP3 variants			Healthy controls		NLRP3 variants		
	Median	Range	Median	Range	*p* value (Mann–Whitney *U* test)	Median	Range	Median	Range	*p* value (Mann–Whitney *U* test)
pRNFL G (μm)	102.0	28.00	101.0	81.00	0.36	99.0	31.0	101.0	8.03	0.20
pRNFL PMB (μm)	53.0	20.00	54.0	52.00	0.86	54.0	24.0	54.5	57.0	0.65
pRNFL N (μm)	76.50	69.00	76.0	87.00	0.43	73.0	69.0	72.5	93.0	0.27
pRNFL T (μm)	66.50	29.00	71.0	73.00	0.62	68.0	29.0	71.0	80.0	0.10
TMV (mm^3^)	2.16	0.30	2.15	0.56	0.93	2.12	0.34	2.14	0.62	0.58
mRNFL (mm^3^)	0.14	0.07	0.14	0.09	0.29	0.13	0.07	0.14	0.10	**0.01**
INL (mm^3^)	0.25	0.14	0.25	0.12	0.53	0.26	0.16	0.25	0.13	0.89
GCIPL (mm^3^)	0.60	0.18	0.59	0.47	0.37	0.59	0.19	0.59	0.50	0.23
OPONL (mm^3^)	0.69	0.22	0.68	0.37	0.84	0.68	0.22	0.68	0.37	0.27

*Note*: Comparison of OCT measures. Data are shown as median and range in μm or mm^3^.

Abbreviations: G, global; GCIPL, combined ganglion cell and inner plexiform layer; HC, healthy control; INL, inner nuclear layer; mRNFL, macular retinal nerve fibre layer; N, nasal; OCT, optical coherence tomography; OPONL, outer plexiform layer and outer nuclear layer; PMB, papillo‐macular bundle; pRNFL, peripapillary retinal nerve fibre layer; T, temporal; TMV, total macular volume.

### Optical coherence tomography changes during follow‐up

Next, group differences during follow‐up visits between HCs and PwNLRP3 were analysed. Average follow‐up time was 2.9 years ±1.4. Only mRNFL thickness (*p* = 0.01) was significantly different in PwNLRP3 in comparison to HCs (see Table 1). No significant differences regarding various other macular and peripapillary layers were observed (global pRNFL, *p* = 0.20; pRNFL papillo‐macular bundle, *p* = 0.65; pRNFL nasal, *p* = 0.27; pRNFL temporal, *p* = 0.10; TMV, *p* = 0.58; INL, *p* = 0.89; GCIPL, *p* = 0.23; OPONL, *p* = 0.27) (see Table 1). Not surprisingly, a subgroup analysis revealed that global pRNFL and GCIPL in patients with a history of ON versus patients without ON differed significantly (global pRNFL [μm], PwNLRP3 without ON 98.85 ± 10.32 vs. PwNLRP3 with ON 86.84 ± 27.70, *p* = 0.0009; GCIPL [mm^3^], PwNLRP3 without ON 0.56 ± 0.11 vs. PwNLRP3 with ON 0.49 ± 0.20, *p* = 0.03).

### Correlation of retinal layers and serum amyloid A levels

Interestingly, mean SAA levels showed a positive association with averaged OPONL volumes (*ρ* < 0.0001, *r* ^2^ = 0.35) (Figure 1g). This effect was not dependent on the presence of MME in the OPONL. The examination of the OPONL revealed no MME in any of the 30 eyes of PwNLRP3. However, microcysts within the INL were observed in 2 out of 30 eyes (n=6.7%). Also associations with other laboratory markers such as CRP and leucocyte counts were not observed. Further analysis of SAA and other retinal layers including INL demonstrated a lack of correlation.

## DISCUSSION

The results of this study provide support that an increase in OPONL volume mirrors concomitant chronic, systemic inflammation in CAPS patients, thereby suggesting OCT as an emerging, candidate biomarker tool for disease monitoring in patients with autoinflammatory syndromes.

It was recently demonstrated that patients with *NLRP3* variants are at particular risk of developing severe CNS inflammation and cranial nerve affection [3, 4, 5]. Of those, ON was the most detrimental manifestation leading to substantial neuroaxonal damage and blindness after multiple and recurrent clinical attacks [10]. Along that line our data indicate a clear retinal neuroaxonal degeneration (global pRNFL and GCIPL) in patients with a history of ON versus patients without ON. These data are consistent with ON manifestation in other chronic inflammatory CNS diseases such as multiple sclerosis [11]. The vulnerability in PwNLRP3 of cranial nerves and especially the proneness of the optic nerve to chronic, sterile inflammation can be readily explained by an increased retinal expression of the *NLRP3* inflammasome [12]. Previous animal studies suggested that optic nerve injuries activate the *NLRP3* inflammasome in retinal microglial cells, typically distributed in GCIPL, OPONL and nerve fibre layers, which then release pro‐inflammatory cytokines, thus further perpetuating neuroinflammation [13, 14]. Vice versa, *NLRP3* knock‐out studies demonstrated a delayed loss and degeneration of retinal ganglion cells following the optic nerve injury [14].

Our study has several limitations including the lack of patients with clear disease‐causing mutations in the *NLRP3* gene, the heterogeneous composition of patients in terms of both clinical characteristics and disease‐modifying therapies and the small sample size, potentially leading to an underestimation of retinal inflammatory effects. Larger and continuous multicentre studies are desired.

Taken together, our study reveals that OPONL and chronic, systemic inflammation in CAPS closely parallel each other over time, suggesting a potential role for OCT as candidate biomarker, not solely for the purpose of tracking PwNLRP3 clinically but also to facilitate treatment decisions. Further multicentre studies with a larger cohort size are required to validate our findings.

## AUTHOR CONTRIBUTIONS

EM: Analysis and interpretation of data, patient care and evaluation, manuscript writing and editing. LB and CT: Acquisition and evaluation of OCT images. MK: Statistical analysis and interpretation of data, manuscript writing and editing. TK: Patient care and contribution to clinical data. JH: Development of study concept, study supervision, patient care and evaluation, analysis and interpretation of data, writing, reviewing and editing of manuscript. All authors discussed the results and commented on the manuscript.

## FUNDING INFORMATION

JH was (partially) funded by the German Federal Ministry of Education and Research (grant numbers 01ZZ1603(A‐D) and 01ZZ1804(A‐H) (DIFUTURE)).

## CONFLICT OF INTEREST STATEMENT

EM and LB report no disclosures. MK received travel funding, speaker honoraria and research support from Bristol Myers Squibb, Merck, Novartis and Roche. TK received travel funding and/or speaker honoraria from Bayer Schering Pharma, Teva, Merck‐Serono, Genzyme, Novartis Pharma, Biogen Idec, received research support from Novartis Pharma. JH reports grants from the Friedrich‐Baur‐Stiftung, Merck and Horizon, personal fees and non‐financial support from Alexion, Horizon, Roche, Merck, Novartis, Biogen, BMS and Janssen, and non‐financial support from the Guthy‐Jackson Charitable Foundation and the Sumaira Foundation.

## Data Availability

The data that support the findings of this study are available from the corresponding author upon reasonable request.
